# Bats in a changing landscape: Linking occupancy and traits of a diverse montane bat community to fire regime

**DOI:** 10.1002/ece3.5121

**Published:** 2019-04-12

**Authors:** Rachel V. Blakey, Elisabeth B. Webb, Dylan C. Kesler, Rodney B. Siegel, Derek Corcoran, Matthew Johnson

**Affiliations:** ^1^ Missouri Cooperative Fish and Wildlife Research Unit, School of Natural Resources University of Missouri Columbia Missouri; ^2^ The Institute for Bird Populations Point Reyes California; ^3^ US Geological Survey, Missouri Cooperative Fish and Wildlife Research Unit, School of Natural Resources University of Missouri Columbia Missouri; ^4^ Forest Service, Plumas National Forest USDA Quincy California

**Keywords:** acoustic, community ecology, ecomorphology, fire ecology, fourth‐corner, RLQ, traits, western United States

## Abstract

1. Wildfires are increasing in incidence and severity across coniferous forests of the western United States, leading to changes in forest structure and wildlife habitats. Knowledge of how species respond to fire‐driven habitat changes in these landscapes is limited and generally disconnected from our understanding of adaptations that underpin responses to fire.
2. We aimed to investigate drivers of occupancy of a diverse bat community in a fire‐altered landscape, while identifying functional traits that underpinned these relationships.
3. We recorded bats acoustically at 83 sites (*n* = 249 recording nights) across the Plumas National Forest in the northern Sierra Nevada over 3 summers (2015–2017). We investigated relationships between fire regime, physiographic variables, forest structure and probability of bat occupancy for nine frequently detected species. We used fourth‐corner regression and RLQ analysis to identify ecomorphological traits driving species–environment relationships across 17 bat species. Traits included body mass; call frequency, bandwidth, and duration; and foraging strategy based on vegetation structure (open, edge, or clutter).
4. Relationships between bat traits and fire regime were underpinned by adaptations to diverse forest structure. Bats with traits adapting them to foraging in open habitats, including emitting longer duration and narrow bandwidth calls, were associated with higher severity and more frequent fires, whereas bats with traits consistent with clutter tolerance were negatively associated with fire frequency and burn severity. Relationships between edge‐adapted bat species and fire were variable and may be influenced by prey preference or habitat configuration at a landscape scale.
5. Predicted increases in fire frequency and severity in western US coniferous forests are likely to shift dominance in the bat community to open‐adapted species and those able to exploit postfire resource pulses (aquatic insects, beetles, and snags). Managing for pyrodiversity within the western United States is likely important for maintaining bat community diversity, as well as diversity of other biotic communities.

## 1 INTRODUCTION

Anthropogenic climate change, drought, fire suppression, and human land use change have led to increases in fire frequency and severity across the globe (Stephens et al., 2014). Changes in fire regime have been particularly evident in the western United States where climate change, land use change, and suppression of more frequent, low severity fires characteristic of historical conditions (Mallek, Safford, Viers, & Miller, 2013; Safford & Stevens, 2017) have led to increases in the number and extent of high severity fires (Dennison, Brewer, Arnold, & Moritz, 2014; Miller & Safford, 2012). With the continued effects of climate change, the western United States is projected to experience 24%–169% increases in annual area burned and an increase in fire season length by 23 days by the mid‐21st century (Yue, Mickley, Logan, & Kaplan, 2013). Although many forests in the western United States are maintained by fire regimes that promote forest heterogeneity (Baker, 2012,2014) and animals have evolved with fire (Pausas & Parr, 2018), comparatively little is known about the implications of rapidly changing fire regimes for managing and maintaining forest biodiversity in the western United States.

Bats are diverse, high trophic level predators that are impacted directly by fire through mortality and injury and indirectly by alteration in roost and foraging habitat availability, and prey communities (Carter, Ford, & Menzel, 2000; Perry, 2012). Fires may influence bat foraging via several mechanisms, mediated by bat ecomorphological traits (Perry, 2012). Fires can substantially alter forest structure by reducing clutter (structurally complex vegetation) and increasing open space, sometimes in the long term (Beaty & Taylor, 2008). Forest structure is an important determinant of insectivorous bat assemblages (Blakey, Law, Kingsford, & Stoklosa, 2017), as bats have diverse morphological and call adaptations for a range of forests from cluttered to open in structure (Schnitzler, Moss, & Denzinger, 2003). For example, a large‐bodied bat with narrow (high aspect ratio) wings and a long duration, low‐frequency call is well adapted to forage on fast prey in open spaces, but has difficulty maneuvering and detecting prey in cluttered habitat (Denzinger & Schnitzler, 2013). In contrast, clutter‐adapted bats can differentiate prey from surrounding vegetation using high frequency, wide bandwidth calls, and maneuver well in small spaces with low aspect ratio wings (Sleep & Brigham, 2003). However, some of these attributes (e.g., slow flight speed) may result in clutter‐adapted bats being relatively more susceptible to predation in open habitats (Lima & O'Keefe, 2013). At a broader landscape scale, fires create high contrast edges, a habitat particularly favored by many foraging bats (Gonsalves, Law, Webb, & Monamy, 2012; Morris, Miller, & Kalcounis‐Rueppell, 2010). Thus, fires that create openings in the landscape and reduce vegetation clutter also may lead to increased foraging opportunities for both open‐ and edge‐adapted foraging bats, while reducing foraging opportunities for bats with clutter‐adapted foraging strategies (Armitage & Ober, 2012; Inkster‐Draper, Sheaves, Johnson, & Robson, 2013). Further, fire can stimulate insect prey production, leading bats to shift foraging behavior to capitalize on abundant postfire insects (Doty, Stawski, Law, & Geiser, 2016; Lacki, Cox, Dodd, & Dickinson, 2009; Malison & Baxter, 2010). Bat adaptations for flight and foraging are likely to influence responses to shifting fire regimes; however, the links between fire regime attributes and bat traits have not been studied.

We evaluated relationships between bat occupancy, bat traits, forest structure, and fire regime in California's Sierra Nevada mountains, where bat diversity is high (17 species; Pierson, Rainey, & Corben, 2001) and frequency and extent of wildfire are increasing (Miller & Safford, 2012). We used a two‐stage approach to investigate relationships between bats and fire. We first tested for relationships between bat occupancy and fire regime and forest structure variables influenced by fire regime. Next, we evaluated ecomorphological traits underpinning these relationships using two trait–environment analyses (fourth‐corner regression and RLQ analysis). We evaluated three fire regime variables in our study: burn severity, years since fire, and fire return interval (FRI). We predicted that bats would show diverse associations to fire regime and forest structure, with open‐ and edge‐adapted bats positively associated with higher severity, more frequent and more recent fires, and clutter‐adapted bats showing the opposite relationship.

## 2 METHODS

### 2.1 Study area

**Figure 1:**
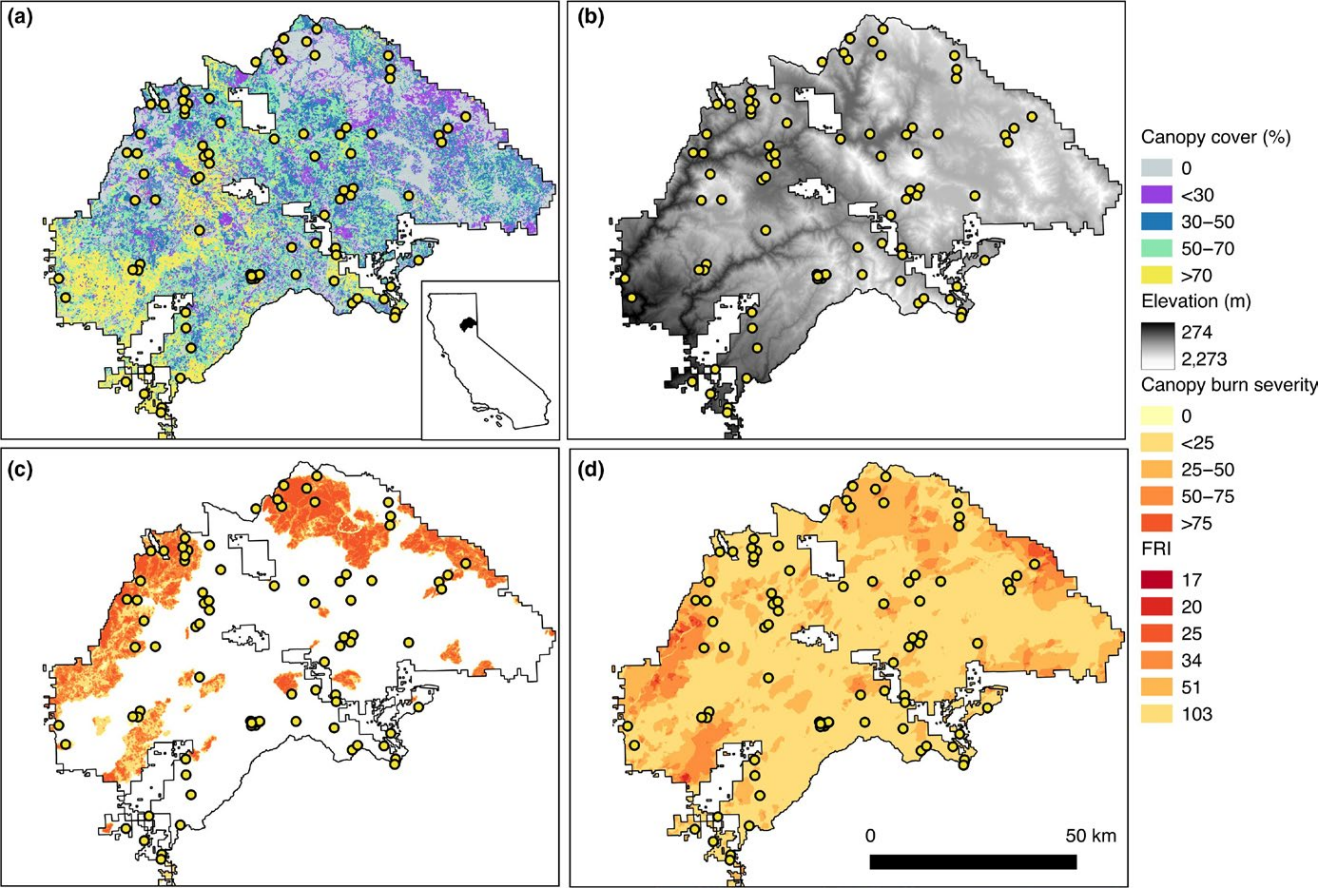
Bats were sampled acoustically at 83 sites (circles) within Plumas National Forest in the Sierra Nevada mountain range, northern California, USA (see inset). Maps show the National Forest boundary with (a) canopy cover (%), (b) digital elevation model (10 m resolution), (c) burn severity (percentage change in canopy cover after fires that burned during 1987–2015), and (d) average fire return interval between 1908 and 2010

We surveyed bats in Plumas National Forest (463,770 ha), within the Sierra Nevada mountain range, in northern California (40°00′01″N 120°40′05″W; Figure 1). The Forest spans an elevation gradient of 311–2,433 m, and has dry and warm summers and cool, wet winters. Mean annual precipitation is high for California (1,036 ± 306 mm), and mean temperature is 10.1 ± 0.9°C., ranging from a mean of 1.3 ± 2.4°C in January to a mean of 19.3 ± 1.5°C in July (1895–2017; Western Regional Climate Center, 2017). Forest communities in Plumas National Forest are dominated by lower and upper montane vegetation such as ponderosa pine (*Pinus ponderosa*) mixed conifer, white fir (*Abies concolor*) mixed conifer, and red fir (*Abies magnifica*), with meadows and montane chaparral present in lower abundance (Fites‐Kaufman, Rundel, Stephenson, & Weixelman, 2007). Additional common tree species include Douglas‐fir (*Pseudotsuga menziesii*), Jeffrey pine (*Pinus jeffreyi*), incense cedar (*Calocedrus decurrens*), and oak (*Quercus* spp.; Fites‐Kaufman et al., 2007). Large‐scale stand‐replacing fires occur regularly in the Plumas National Forest, with five extensive (>20,000 ha) fires within the last 20 years (USDA Forest Service, 2017).

### 2.2 Bat surveys

We randomly selected sampling sites across Plumas National Forest, after removing areas with slopes >15% to improve accessibility of sites (7.8% of the study area). We sampled each site over three consecutive nights, recording echolocating bats using Pettersson D500x bat detectors (Pettersson Elektronik, Uppsala, Sweden). Surveys took place over portions of three summers, corresponding to bat lactation season and the period of greatest bat activity (8 June–31 July 2015, 31 May–8 August 2016, and 12 June–24 July 2017), and individual sites were not revisited in multiple years. At each site, we placed one bat detector with its microphone in a weatherproof PVC tube angled 90° from vertical at a height of 2 m. Detectors were programed to sample from before sunset to after sunrise (7:30p.m.–6:30a.m. Pacific Daylight Time) for three consecutive nights and we did not sample during rainfall or strong wind. We used automated acoustic analysis software (*SonoBat 4.2.2*, SonoBat, Arcata, CA, US) to identify recorded calls to species where possible. We used the *SonoVet* tool to manually check calls that had been identified to species by the software to ensure a high level of confidence in identification (Russo & Voigt, 2016). If calls identified to species were not of sufficient quality with features that enabled us to confidently separate them from similar species, that species was not recorded as present for that night at that site. While this approach may increase the probability of false negatives, it reduces false positives, which are more problematic for model inference (Miller et al., 2011). Our final bat dataset spanned 249 survey nights (83 sites for three nights each) and included detections of all 17 bat species known to occur in the region.

### 2.3 Nonfire environmental variables

We used three types of nonfire environmental variables in our occupancy models: detection covariates, physiographic variables, and forest structure variables. Detection covariates included variables that were likely to influence nightly fluctuations in bat activity: nightly weather (minimum and maximum temperature) and moon phase (illuminated fraction and moon illuminance; Saldaña‐Vázquez & Munguía‐Rosas, 2013; Turbill, 2008). Physiographic (elevation, slope, distance to water, and percent rock cover) and forest structure (canopy cover, mean tree diameter, stand basal area, trees per ha, distance to open area, and distance to forest edge) variables were used to characterize habitat relationships for each bat species, prior to explicitly examining effects of fire. For field measurements, site was defined as a 100 by 100‐m plot centered on the bat detector, encompassing the likely range of detection distances for bats in the study area.

We used the closest three National Oceanic and Atmospheric Administration weather stations to each site (with a maximum distance of 40 km) to measure daily maximum and minimum temperature. We used the *rnoaa v0.7.0* R package to access climate data (Chamberlain et al., 2017). We avoided sampling on rainy nights; precipitation was recorded during seven (8%) sampling periods only with one sampling night recording >5 mm rainfall (9.8 mm). Moon illuminance and fraction illuminated were calculated with methods described by Upham and Hafner (Upham & Hafner, 2013) using the US Naval Observatory Multi‐year Interactive Computer Almanac (MICA) v2.2.2 (United States Naval Observatory, 2011) and the *oce v0.9‐20* R package (Kelley, Richards, & Layton, 2016).

We extracted elevation at each site from a 10 m digital elevation model (US Geological Survey, 2017) and calculated slope using the eight pixels surrounding each site location using the *raster v 2.6‐7* package in R (Hijmans et al., 2017). We characterized water availability by extracting the distance to water bodies and perennial streams using the NHD Plus v2 dataset (McKay et al., 2012). Overall, we included four water variables: distance to water body (mapped lakes, wetlands, dams, and reservoirs), distance to perennial stream, distance to (any) stream, and distance to (any) water (all aforementioned). We estimated percent rock cover at each site within a 100 by 100‐m plot centered on each bat detector as the average percentage of exposed rock along four transects, starting at the detector and extending for 50 m in each cardinal direction. In the same plots, we estimated canopy cover using a concave densitometer directly above the detector and at four locations 50 m from the detector in each cardinal direction; the five densitometer measurements were averaged at each site. We used a wedge prism to count and estimate the diameter at breast height (DBH) of standing (live or dead) trees within 50‐m of the detector. To partially account for annual and seasonal variation in occupancy (due to breeding phenology and/or migration), we included day of season (1–71), starting with the earliest date of bat recordings across the three seasons, 3 May (1), up to the latest date of bat recordings across the three seasons, 9 August (71), as well as survey year.

### 2.4 Fire regime variables

We included three variables describing variation in fire regime in our study area: years since fire, fire return interval, and canopy burn severity. We used current fire return interval (FRI; Safford, VandeWater, & Clark, 2016), calculated by dividing number of years the dataset spanned (107 years, 1908–2015) by the number of fires in that period plus one. As there were only three values of FRI within our sites, we treated this variable as both a continuous and categorical variable (frequent = 36, regular = 54 and rare = 107 years). Years since fire was also obtained from Safford et al. (2016), with areas that never burned during the 107‐year period allotted the value 107 (values ranged from 3 to 107 years). Vegetation burn severity calibrated to percent change in canopy cover (hereafter: “burn severity”) was obtained from another USDA Forest Service vector product (USDA Forest Service, 2017) for all fires in the study area between 1987 and 2017, and was estimated using methods described in Miller and Thode (2007). The burn severity product included six categories of percent change in canopy cover, 0 = unburned, 1 = burned but no change in canopy, 2 = <25%, 3 = 25%–50%, 4 = 50%–75%, 5 = >75% burned. As there were not sufficient spread of data across categories (e.g., burn severity of three only had three values) to treat this as a categorical variable, we calculated means of the percentage ranges and treated it as a continuous variable (0, 12.5, 37.5, 62.5, 87.5). Burn severities were unavailable for six of 83 sites that burned between 34 and 89 years prior to the study and were treated as having a burn severity of zero. Although there were more unburned (*n* = 51) than burned (*n* = 32) sites in our study and sites were not stratified across fire regime or forest structure variables, we believe variability across the study landscape was sufficient for testing relationships. Five small fires burned within Plumas National Forest during the study (2015–2017), which we consider unlikely to affect study findings given these fires were not close to sampled sites (the closest site was 6 km to one of the burned areas) and covered a small proportion of the study area (<2%). Years since fire and FRI were highly correlated (*R* = 0.95), whereas burn severity was moderately negatively correlated with TSLF (−0.69) and FRI (−0.61).

### 2.5 Relationships between fire regime, forest structure, and bat species occupancy

We used single‐season occupancy modeling (MacKenzie et al., 2002) and an information theoretic approach to model selection (Burnham & Anderson, 2002) to evaluate predictors of bat occupancy. We first identified the physiographic and forest structure variables influencing occupancy for each species, comparing models of all possible combinations of uncorrelated physiographic and forest structure variables, ranked by AICc values. We also evaluated whether temporal variation among periods influenced bat occupancy probability by including two covariates in the model selection process (day of season and year). We avoided collinearity by including only the best‐fitting variable among correlated sets (e.g., distance to waterbody, perennial, stream, and water). Initial best models were selected for each species, retaining the highest ranking model based on Akaike's information criterion adjusted for sample sizes (AICc). Next, we identified detection covariates important for each bat species by comparing these initial best models plus all combinations of detection covariates, retaining the model with the combination of detection covariates that minimized AICc. Moon fraction and moon illuminance were correlated (*R* = 0.75), thus we only included the variable more strongly correlated with detection for each species. The resulting top‐ranking models containing detection and site covariates were considered “biological null models.” Next, we assessed whether bat occupancy was influenced by fire regime by adding each of the three fire regime variables, separately, to the base model. For years since fire, both linear and curvilinear relationships were plausible so we evaluated both linear and quadratic (2nd order polynomial) fits and retained the variable with the lowest AICc. For FRI, we also retained the variable (continuous or categorical) with the lowest AICc. For each species, we then had four models. The biological null models contained only detection, physiographic, and forest structure variables (nonfire regime variables). Three separate models contained the base model and one of the fire regime variables, which included: burn severity, years since fire (either linear or quadratic fit), and FRI (either continuous or categorical). Fire variables were fit separately rather than together, given correlations among fire variables. We ranked the four models for each species and retained the top‐ranking model using AICc as the full model. Where fire models did not outcompete biological null models by >2 ΔAIC*_c_*, the fire variable was considered uninformative and the biological null model was retained as the full model (Arnold, 2010). Here and elsewhere, we considered differences statistically significant at *α* < 0.05. The fit of final models were checked using Dunn–Smyth residuals (Warton, Stoklosa, Guillera‐Arroita, MacKenzie, & Welsh, 2017) and using a parametric bootstrap (*n* = 10^4^ bootstraps) of Pearson's chi‐square test (MacKenzie & Bailey, 2004). We used the *unmarked v0.12‐2* package (Fiske & Chandler, 2011) to fit occupancy models and the *MuMIn v1.40.4* package (Barton, 2015) for model selection.

### 2.6 Relationships between fire regime, forest structure, and bat traits

For each of the 17 bat species detected, we compiled data on five “ecomorphological” traits likely to correspond with adaptations to different forest structure and fire regime variables (Table 1). Call traits included characteristic call frequency (Fc), call bandwidth (BW), and call duration (Dur) and were taken from summaries of western United States bat call characteristics included in Sonobat (*SonoBat 4.2.2*, SonoBat, Arcata, CA, US). We used one morphological trait (body mass in g), as previously published estimates were available for all species in our study, and body mass is highly correlated with other morphological traits such as forearm length (mm; *R* = 0.95), wing loading (*R* = 0.97), and wing aspect ratio (*R* = 0.87; sources listed in Table 1). We categorized foraging strategy into one of three broad groups: open‐adapted foragers, edge‐adapted foragers, and clutter (structurally complex vegetation)‐adapted foragers, based on previously reported foraging behavior (sources listed in Table 1). While these foraging strategies are broad classifications that have been found to be strongly related to three‐dimensional forest structure (Blakey et al., 2017), they do not preclude bats from using a variety of structures while foraging (Denzinger & Schnitzler, 2013).

**Table 1:** Bat species detected in Plumas National Forest, Sierra Nevada, CA, with percentage of the 83 sites in which each species was recorded (%), total nights detected (*n*), and mean detection (*ρ*) and occupancy (*ψ*) probabilities for top‐ranked models (Appendices S1 and S2)

Scientific name	Common name	Sp code	%	*n*	*ρ*	*ψ*	Fc kHz	BW kHz	Dur ms	Mass g	Foraging strategy
*Myotis californicus*	California myotis	Myca	83	160	0.77 ± 0.03	0.92 ± 0.04	49.1	54.3	3.8	4.2	Edge (Frick, Hayes, & Heady, 2009)
*Myotis evotis*	Western long‐eared myotis	Myev	74	114	0.58 ± 0.04	0.89 ± 0.05	34.3	50.4	3.7	7.3	Clutter (Faure, Fullard, & Barclay, 1990)
*Lasionycteris noctivagans*	Silver‐haired bat	Lano	53	92	0.67 ± 0.05	0.58 ± 0.07	26.5	16.1	9.2	10.6	Edge (Barclay, 1986)
*Eptesicus fuscus*	Big brown bat	Epfu	42	66	0.55 ± 0.06	0.53 ± 0.01	28.2	29.4	7.8	15.9	Edge (Frick et al., 2009)
*Tadarida brasiliensis*	Mexican free‐tailed bat	Tabr	37	59	0.55 ± 0.17	0.43 ± 0.08	25.5	8.2	11.5	12.5	Open (Frick et al., 2009)
*Lasiurus cinereus*	Hoary bat	Laci	29	32	0.27 ± 0.06	0.47 ± 0.12	20.1	6.3	11.0	33.0	Open (Barclay, 1986)
*Myotis lucifugus*	Little brown bat	Mylu	29	48	0.46 ± 0.08	0.29 ± 0.09	40.8	36.4	6.0	7.1	Edge (Burles, Brigham, Ring, & Reimchen, 2008; Ratcliffe & Dawson, 2003)
*Myotis thysanodes*	Fringed myotis^b^	Myth	24	30	0.38 ± 0.08	0.26 ± 0.08	24.5	52.6	3.9	8.4	Clutter (O'Farrell & Studier, 1980)
*Myotis yumanensis*	Yuma myotis	Myyu	19	28	0.29 ± 0.08	0.25 ± 0.08	49.2	44.4	5.5	5.2	Edge (Frick et al., 2009)
*Antrozous pallidus*	Pallid bat^a^, ^b^	Anpa	19	21	NA	NA	28.0	28.3	6.8	17.3	Clutter (Frick et al., 2009)
*Myotis volans*	Long‐legged myotis	Myvo	18	18	NA	NA	41.6	52.7	4.8	10.4	Edge (Frick et al., 2009)
*Lasiurus blossevillii*	Western red bat^a^	Labl	10	13	NA	NA	38.9	15.8	10.7	12.5 (Harvey, Altenbach, & Best, 2011)	Edge (Frick et al., 2009)
*Corynorhinus townsendii*	Townsend's long‐eared bat^a^, ^b^	Coto	5	6	NA	NA	23.4	21.1	4.6	10.2	Clutter (Fellers & Pierson, 2002; Segura‐Trujillo, Lidicker, & Álvarez‐Castañeda, 2016)
*Myotis ciliolabrum*	Small‐footed myotis	Myci	4	5	NA	NA	44.3	54.5	3.2	4.9 (Barclay & Brigham, 1991)	Edge (Holloway & Barclay, 2001)
*Parastrellus hesperus*	Canyon bat	Pahe	5	5	NA	NA	45.9	15.2	5.5	4.4	Edge (Segura‐Trujillo et al., 2016)
*Eumops perotis californicus*	Western mastiff bat^a^	Eupe	2	3	NA	NA	10.4	10.4	15.4	53.5	Open (Best, Kiser, & Freeman, 1996)
*Euderma maculatum*	Spotted bat^a^	Euma	2	2	NA	NA	10.0	4.9	3.2	17.9 (Barclay & Brigham, 1991)	Clutter (Segura‐Trujillo et al., 2016)

Note

Sparse detections for eight species precluded modeling detection and occupancy probabilities. Call traits included characteristic call frequency (Fc), call bandwidth (BW), and call duration (Dur) and were obtained from summaries of western United States bat call characteristics included in Sonobat (*SonoBat 4.2.2*, SonoBat, Arcata, CA, US). We used body mass from the *PanTHERIA* database (Jones et al., 2009) except where indicated and foraging strategy was taken from multiple sources.

a California Species of special Concern by California Department of Fish and Wildlife.

b USDA Forest Service, Pacific Southwest Region, 2013, Regional Forester's Sensitive Species (https://www.fs.usda.gov/detail/r5/plants-animals/wildlife/).

We investigated consensus between two methods to identify how bat ecomorphological traits related to forest structure and fire regime variables: a model‐based fourth‐corner analysis (Brown et al., 2014; Legendre, Galzin, & Harmelin‐Vivien, 1997) and an ordination‐based RLQ analysis (Dolédec, Chessel, Ter Braak, & Champely, 1996). These methods allowed us to explore specific ecomorphological traits underpinning species–environment relationships. Both methods used three matrices: environmental data for each site (R), species occurrence for each site (L), and species' traits (Q). The model‐based fourth‐corner analysis predicted presence (species recorded during at least one sampling night), as a function of explanatory variables (forest structure and fire regime variables), species traits and the interaction between environmental variables and species traits (Brown et al., 2014). The coefficients for the interaction between environmental variables and species traits were the “fourth‐corner” terms (Brown et al., 2014), allowing quantification of specific trait–environment relationships. As these methods do not allow for repeated sampling, we converted our data to presence–absence format, by assigning sites where a species was recorded during at least one night as one, and coding sites where the species was never recorded over the three nights as zero. While we recognize that “absences” may depict a lack of detection rather than true absences, mean detection probabilities were high (>50%) making it likely that most species were detected within the 3‐night period (Table 1). We used the binomial family to fit the models and employed a LASSO penalty (Hastie, Tibshirani, & Friedman, 2009) for automatic model selection, which removed all fourth‐corner interactions not improving model fit (Brown et al., 2014). We used the *mvabund v.3.13.1* package to fit fourth‐corner regression models (Wang, Naumann, Wright, & Warton, 2012).

The RLQ analysis approaches the same “fourth‐corner” problem by performing a simultaneous ordination of the three matrices, producing an overview of trait–environment relationships, visualized in scores (eigenvalues) across two axes (Dray et al., 2014). The strength and direction of associations between traits and environment (forest structure and fire regime variables) can be interpreted from the size and sign of the eigenvalue. We modeled a set of uncorrelated (|*R*| < 0.7; Dormann et al., 2013) forest structure and fire regime variables including the following: mean tree diameter, tree basal area, trees per ha, canopy cover, burn severity, and FRI. To test significance of trait–environment relationships, we used an analysis of deviance with row‐resampling and 10^4^ bootstrap iterations for the fourth‐corner regression and a Monte Carlo permutation test with 10^6^ iterations for the RLQ analysis. We used the *ade4 v1.7‐10* package to perform RLQ analysis (Dray & Dufour, 2007).

## 3 RESULTS

### 3.1 Relationships between fire regime, forest structure, and bat species occupancy

**Figure 2:**
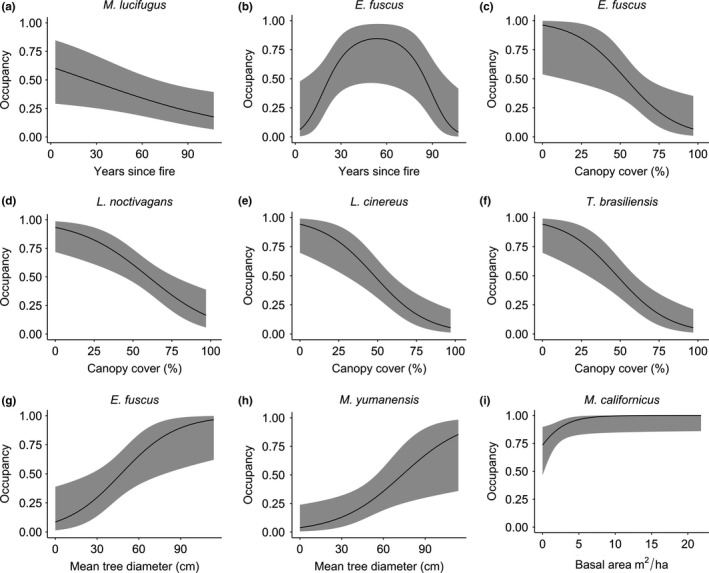
Relationships between forest structure and fire regime variables (Appendix S2) and predicted occupancy probabilities of seven bat species in the Sierra Nevada, California. Models are single‐season occupancy models and fitted lines are shown with 95% confidence intervals (shaded area). All other covariates (aside from the focal variable) were fixed to mean values to produce the figures presented herein. All relationships plotted were statistically significant (*α* < 0.05)

During 249 recording nights (June‐August, 2015–2017), we documented 17 bat species, including five listed as species of special concern by the California Department of Fish and Wildlife (*Antrozous pallidus*,* Corynorhinus townsendii*, *Euderma maculatum*,* Lasiurus blossevillii*, and* Eumops perotis californicus*; California Department of Fish & Wildlife Natural Diversity Database, 2017) and three designated as Forest Service sensitive species (*A. pallidus*,* C. townsendii*, and* Myotis thysanodes*; Table 1). For nine species with sufficient detections to allow modeling of occupancy and detection probabilities (detected >10% of nights), final models of three species contained fire regime variables (*Myotis evotis*, *Myotis lucifugus*, *Eptesicus fuscus*: years since fire; Appendices S1 and S2). Of these, *M. lucifugus* and *E. fuscus* showed a statistically significant relationship with years since fire (Figure 2a,b, Appendices S1 and S2). Probability of *M. lucifugus* occupancy decreased with years since fire (Figure 2a), whereas *E. fuscus* occupancy probability increased with years since fire until approximately 54 years, and then began to decrease (Figure 2b).

Forest structure variables were important predictors of bat species occupancy, with 7 of 9 species related to at least one forest structure variable (Appendix S2). Probability of occupancy decreased with increasing canopy cover for two edge‐adapted and two open‐adapted species (*E. fuscus*, *Lasionycteris noctivagans*,* Lasiurus cinereus*, and *Tadarida brasiliensis*, respectively; Figure 2c–f; Appendix S2). Basal area was positively associated with an edge‐adapted bat (*Myotis californicus*; Figure 2i; Appendix S2), and mean tree diameter was positively related to occupancy probability for two edge‐adapted species: *Myotis yumanensis* and *E. fuscus* (Figure 2g,h; Appendix S2). Among the physiographic variables, *M. evotis *and *M. lucifugus* were more likely to occur at higher elevations, *M. californicus *at lower elevations and *M. thysanodes* more likely to occur on gentler slopes (Appendix S2). For seven of nine species, daily maximum temperature was a predictor of bat detection probability, with greater detection probabilities at higher temperatures (Appendix S2). Day of season was not a predictor of occupancy probability for any of the species; however, year was a predictor of occupancy of *L. cinereus*, with occupancy probability higher in 2015 than in 2016, though no other pairwise comparisons were significant (Appendix S2).

### 3.2 Relationships between fire regime, forest structure, and bat traits

**Figure 3:**
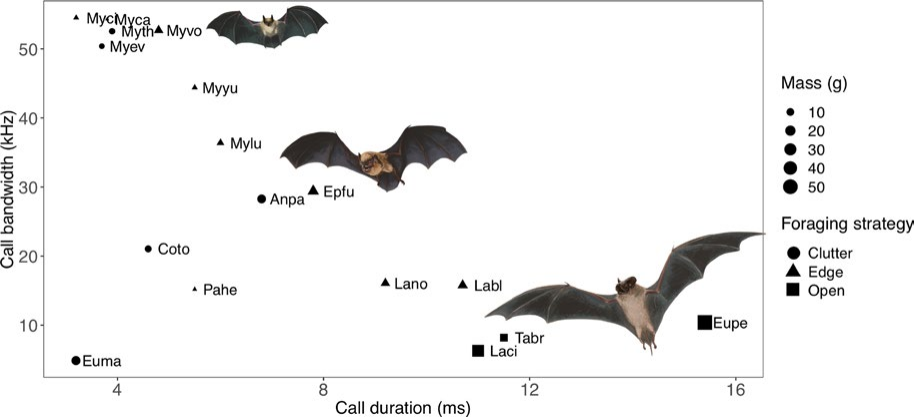
Four ecomorphological traits for 17 bat species detected in Plumas National Forest, based on values from the literature (see Table 1). Squares represent open‐adapted, triangles depict edge‐adapted and circles represent clutter‐adapted bat species. Species codes are given in Table 1

Traits of the 17 bat species in the study area showed trends consistent with ecomorphological theory. Bats with lower frequency calls generally also had narrower call bandwidth, longer call duration, and larger body mass, with open‐adapted foraging strategies more likely (Figure 3). Clutter‐adapted bats varied in call frequency, bandwidth, and body mass, but all had relatively short call duration (Figure 3). Edge‐adapted bats also showed variation in call traits; however, all had body mass below 16 g (Figure 3) and call frequencies above 25 kHz.

**Figure 4:**
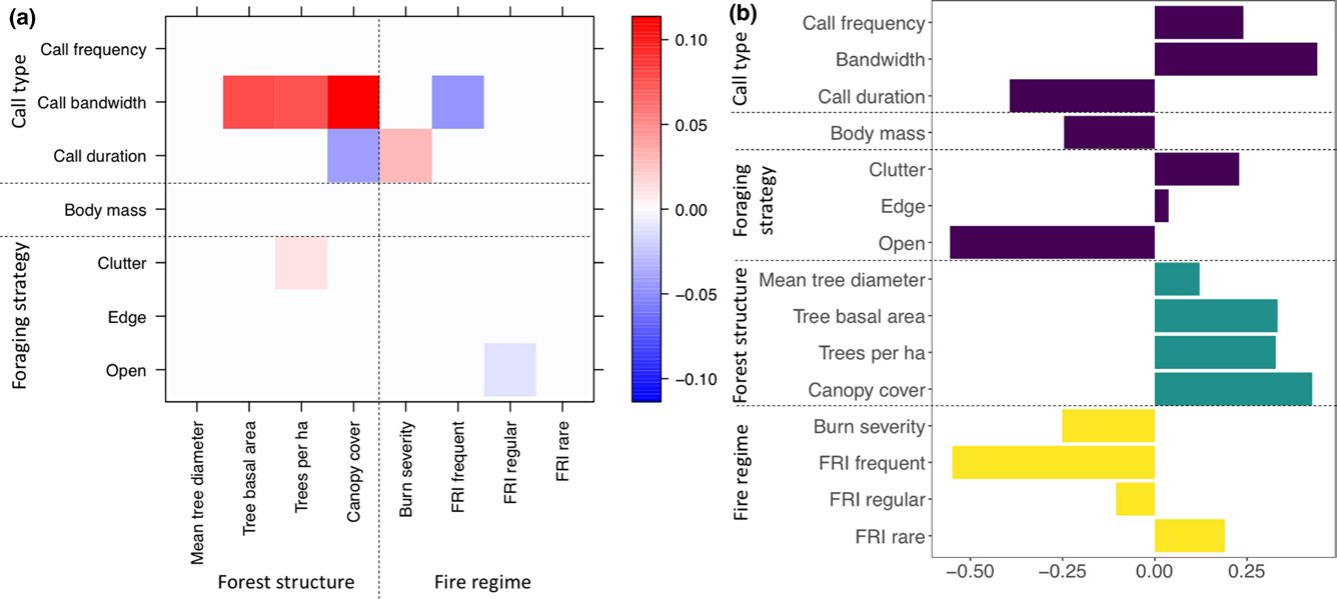
Relationships between 5 traits of 17 bat taxa and four forest structure and two fire regime variables based on (a) fourth‐corner regression and (b) RLQ analysis. In the fourth‐corner regression (a), the strength and direction of relationships between traits (*y*‐axis) and forest structure and fire regime variables (*x*‐axis) is indicated by color (red: positive and blue: negative). For example, in the fourth‐corner regression (a), bats with longer duration calls are associated with higher burn severity. In the RLQ analysis (b), the *x*‐axis shows eigenvalues for Axis 1 and the *y*‐axis shows five bat traits (purple), four forest structure variables (green), and two fire regime variables (yellow). The strength and direction of Axis 1 eigenvalues of the RLQ analysis (b) indicate how traits covary with forest structure and fire regime variables. For example, in the RLQ analysis (b) open‐adapted bats were most strongly associated with frequent fires (FRI frequent)

Traits of 17 bat species were related to fire regime and forest structure variables based on our fourth‐corner regression model (Deviance = 90.95, *p* < 0.0001) and the RLQ (Monte Carlo permutation test: *p* < 0.0001). The first axis of the RLQ analysis (Figure 4b) explained 97.8% of the co‐structure of traits with forest structure and fire regime variables. Strength of predictors of the trait‐environment relationship is indicated by number of associations and darkness of color in the fourth‐corner analysis (Figure 4a) and magnitude of axis 1 eigenvalue (*x*‐axis) in the RLQ analysis (Figure 4b). Strongest predictors of the trait‐environment relationships among forest structure and fire regime variables were canopy cover, trees per ha, tree basal area, burn severity and FRI, while strongest trait predictors were call bandwidth, call duration and foraging strategy (Figure 4).

**Figure 5:**
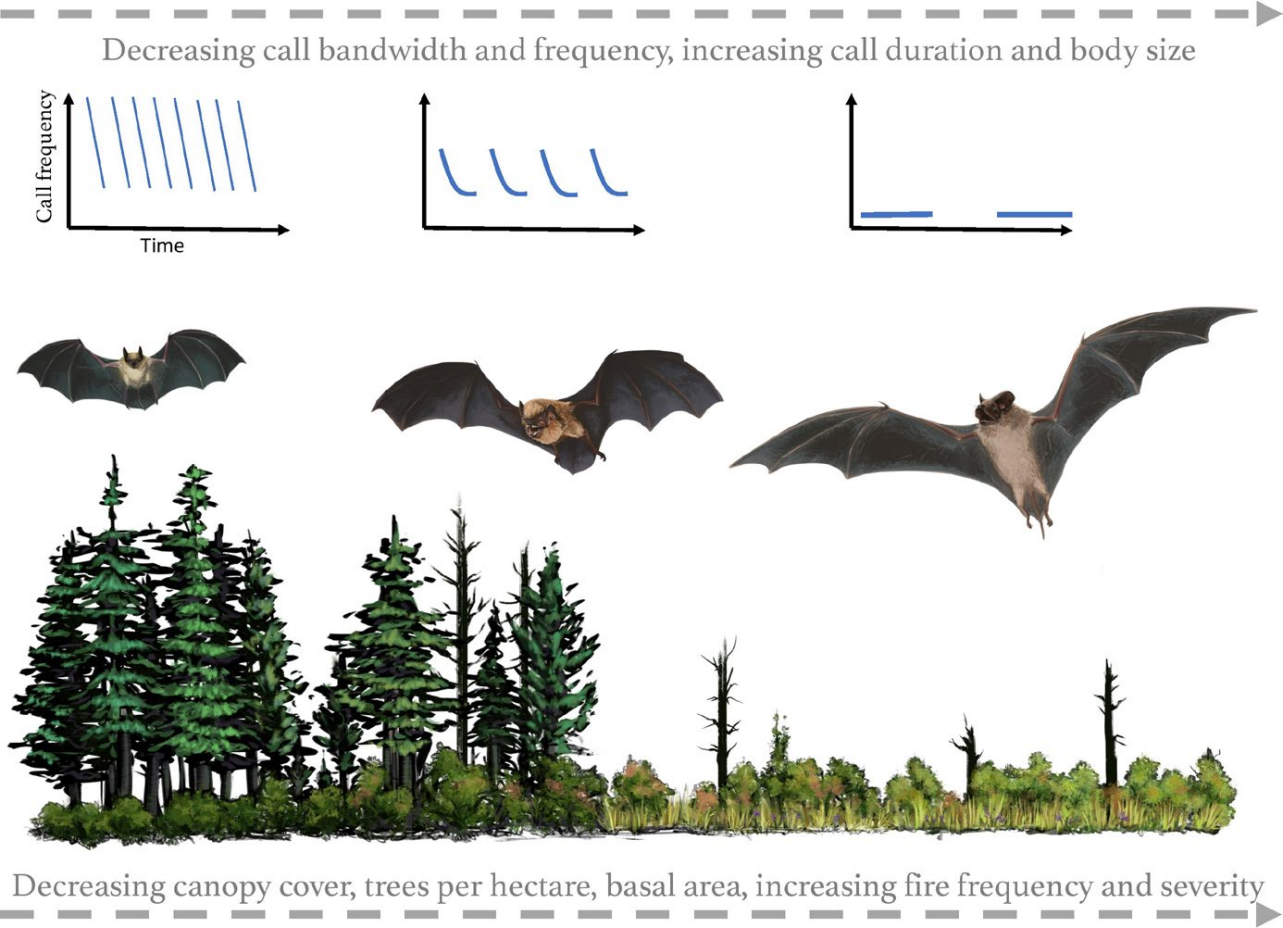
Illustration of ecomorphological relationships revealed in this study. As habitats change across a gradient of increasing burn severity and frequency and decreasing clutter (left to right), larger bats with narrower bandwidth, lower frequency and longer duration calls are more likely to occupy the area. From left to right, representatives from three bat foraging strategies are shown: clutter‐adapted (*Myotis thysanodes*), edge‐adapted (*Eptesicus fuscus*), and open‐adapted (*Tadarida brasiliensis*). Body sizes are not to scale

Overall, relationships between traits and forest structure were consistent with predictions based on bat ecomorphology (Figure 5). The fourth‐corner analysis (Figure 4a) indicated bats with broader call bandwidth and, to a lesser extent shorter calls and clutter‐adapted foraging strategy, were associated with more cluttered sites (higher canopy cover, basal area and trees per ha or mean tree diameter; Figure 4a). All of these relationships were supported by the RLQ analysis, which further indicated open‐adapted bats with greater body mass and lower call frequencies were negatively associated with forest clutter (Figure 4b). Canopy cover, trees per ha and tree basal area showed the strongest trait‐environment relationships across both analyses, with mean tree diameter showing the weakest relationships with bat traits (Figure 4). There was some consensus between both analyses in the relationships between bat traits and fire regime; bats with longer duration calls were associated with greater burn severities and narrower bandwidth calls were associated with more frequent fires. The weaker negative relationship between open‐adapted foraging bats and regularly occurring fire in the fourth‐corner analysis was not supported by the RLQ analysis, which showed a weak positive relationship between open‐adapted bats and regular fire (Figure 4b). Relationships between bat traits and fire identified by the RLQ analysis, but not the fourth‐corner analysis, included a positive association between open‐adapted bats, frequent fire, and burn severity, and a negative relationship for clutter‐adapted bats. There was no evidence for relationships between edge‐adapted foraging bats and fire or forest structure in either analysis (Figure 4). In the RLQ, rare fires were associated with cluttered forest structure, while frequent fires with higher burn severity were associated with more open forest structure (Figure 4b).

## 4 DISCUSSION

Results indicate variable responses to fire regime and forest structure within a diverse montane forest bat community, underpinned by ecomorphological traits of individual species. Bat adaptations to forest structure largely explained the relationships between fire regime and bat traits. Greater burn severities, more frequent fires, and hence more open forests (lower canopy cover and basal area) favored bats with traits adapting them to foraging in open habitats, consistent with previous research (Armitage & Ober, 2012; Buchalski, Fontaine, Heady, Hayes, & Frick, 2013; Cox, Willcox, Keyser, & Vander Yacht, 2016; Inkster‐Draper et al., 2013). Conversely, our trait–environment analysis indicated that bats with clutter‐adapted traits were negatively associated with burn severity and frequency of fires, although the literature reports positive or no effect of burn severity (Buchalski et al., 2013; Lacki, Dodd, Skowronski, Dickinson, & Rieske, 2017) and no effect of fire frequency (Armitage & Ober, 2012). These patterns mirrored results from bird studies in which aerial insectivores adapted to open and edge habitats are more abundant in burned forest while gleaning species (adapted to cluttered habitats) are more abundant in unburned forest (Kotliar et al., 2002) and are negatively associated with burn severity (Azeria et al., 2011).

Relationships between fire and individual species occupancy were neutral or positive in our study, consistent with other studies of effects of wildfire on bat activity in United States and Australia (Buchalski et al., 2013; Law, Doty, Chidel, & Brassil, 2018). Low correlation between edge‐adapted foraging strategy and fire and forest structure variables (Figure 4) was likely due to variation in individual species' relationships to fire regime and forest structure variables (Figure 2). Previous studies of *M. lucifugus* and *E. fuscus* reported no effect of fire on activity (Austin, Silvis, Ford, Muthersbaugh, & Powers, 2018; Loeb & Waldrop, 2008; Silvis, Gehrt, & Williams, 2016), but that work focused on prescribed fire, which likely burned at relatively lower severity than much of the wildfire that occurred within our study area. The positive association between *M. lucifugus* occupancy and recent fires may be influenced by the species' preference for aquatic prey, as pulses in aquatic productivity can be stimulated by fire (Malison & Baxter, 2010; Roby & Azuma, 1995). Varying effects of years since fire and forest structure on edge‐adapted bats like *E. fuscus* may reflect responses to larger scale configuration of fire habitat, for example, availability and juxtaposition of edges and forest openings (Loeb & O'Keefe, 2011; Morris et al., 2010). Adding to the complexity of edge‐adapted bats' relationships with fire, a study of bat activity (rather than occupancy) indicated increased use of habitats with greater burn severities in the Sierra Nevada for several edge and clutter‐adapted bats including medium‐high frequency calling *A. pallidus*, *M. thysanodes*, and undifferentiated *Myotis *spp. (Buchalski et al., 2013). Whereas generalizations can be made between adaptations to forest structure and effects of fire regime in the western United States, the variable relationships of edge‐adapted bat species to fire regime variables may indicate that unexplored complexities in the relationships persist. Alternatively, given edge‐adapted foragers have fewer specialized traits, when compared to open‐ and clutter‐adapted bats, they may employ more flexible foraging strategies and adapt to a greater variety of conditions (Denzinger & Schnitzler, 2013). Finally, study scale and observation error associated with fire regime variables may obscure existing relationships between bats and burned landscapes.

Predicted increases in the number and extent of high severity fires in coniferous forests of the western United States (Dennison et al., 2014; Miller & Safford, 2012) are likely to increase foraging opportunities for open‐ and edge‐adapted bats with relatively longer duration and narrower bandwidth calls (e.g., *E. perotis*, *L. cinereus*, *T. brasiliensis*, *L. noctivagans*). However, increases in fire frequency and severity may reduce foraging opportunities for clutter‐adapted bats and bats with shorter duration and wider bandwidth calls (e.g., *C. townsendii*, *M. evotis*, *M. thysanodes*, *Myotis ciliolabrum*, *Myotis volans*, *M. californicus*), including species that are most at risk from the spread of white‐nose syndrome in the Western United States (Weller et al., 2018). Increasing fire frequency and severity could have positive short‐term (5 years postfire) effects for bats that forage on aquatic insects (Malison & Baxter, 2010) such as *M. lucifugus* and *M. yumanensis* via increased postfire pulses of aquatic productivity. Similarly, the primary prey for *E. fuscus*, *A. pallidus,* and *Parastrellus hesperus* are beetles, which are abundant in postfire landscapes (Kral, Limb, Harmon, & Hovick, 2017). However, moths (Lepidoptera) are the primary prey of the remaining 12 species in our study and may be negatively affected by fire (Armitage & Ober, 2012; Kral et al., 2017). *Myotis evotis*, the only species in our study area for which roosting preferences with relation to fire have been studied, selected roosts away from burned areas (Snider, Cryan, & Wilson, 2013). High severity fire destroys canopy, hence reducing roost availability for foliage roosting species like *L. cinereus* and *L. blossevillii*; fire may also cause direct mortality of these species if it occurs during winter when they may roost within leaf litter (Johnston & Whitford, 2009; Perry & McDaniel, 2015). However, many of the species in our study area use snags, hollows, crevices, and exfoliating bark to roost, structures that may be created or enhanced by fire (Johnson, Edwards, Ford, & Gates, 2009; O'Keefe & Loeb, 2017).

Bat community diversity in the increasingly fire‐prone forests of the western United States likely will benefit from management for mixed‐severity fire and pyrodiverse landscapes, which also has been shown to be important for bird communities in the region (Kelly & Brotons, 2017; Tingley, Ruiz‐Gutiérrez, Wilkerson, Howell, & Siegel, 2016). Variable severity fire creates a mosaic of forest gaps and edges (Comfort, Clark, Anthony, Bailey, & Betts, 2016), which are high‐quality foraging habitat for many bat species (Gonsalves et al., 2012; Loeb et al., 2011; Morris et al., 2010) including the 12 open‐ and edge‐adapted species in our study as well as some clutter‐adapted bats. Low severity fire may promote growth of larger trees, by thinning smaller trees (Brown, Mutch, Spoon, & Wakimoto, 1995), and may increase habitat quality for small (*M. californicus* and *M. yumanensis*) and medium‐sized (*E. fuscus*) edge‐adapted bats (Figure 2, Appendix S2). Years since fire is also an important predictor of bat occupancy in our study area (Figure 2, Appendix S2). Natural postfire succession following variable severity fire facilitates development of structurally heterogeneous environments at the landscape scale with pockets of cluttered forest interspersed, and initial (<15 years) natural regeneration after high severity fire in the Sierra Nevada creates heterogeneous land‐cover patterns (Hanson, 2018). However, rarely burned areas also are important for clutter‐adapted bats in our study area, four of which are of conservation concern (Table 1). Pyrodiversity also is likely to promote bat prey diversity (Kral et al., 2017) and roosting opportunities (Johnson et al., 2009; O'Keefe & Loeb, 2017). Managing for diverse fire regimes, at the appropriate scale, facilitates habitat development and maintenance for species where roosting and foraging habitats diverge (Azeria et al., 2011). Although pyrodiversity does not always lead to increased wildlife biodiversity (Pastro, Dickman, & Letnic, 2011; Taylor et al., 2012), it is likely to benefit wildlife including bats, birds, and insects within the fire‐prone forests of the western United States (Buchalski et al., 2013; Ponisio et al., 2016; Tingley et al., 2016). Management strategies aiming for mixed‐severity fire also are more likely to be consistent with historical fire regimes in the Western United States (Baker, 2014).

A better understanding of the links between fire regimes and wildlife traits can aid in the development and selection of management options that maximize biodiversity in the fire‐prone forests of the western United States. These forests host diverse animal assemblages, adapted to a variety of fire‐mediated habitat conditions (Buchalski et al., 2013; Roberts, Kelt, Van Wagtendonk, Miles, & Meyer, 2015; Rochester et al., 2010; White et al., 2016), making it challenging for forest managers to decide where to allocate limited management resources. Identifying relationships between fire regime and species functional traits allows forest managers to: a) identify which fire characteristics or treatments are most important for particular species and broader communities; b) manage forests with fire for large numbers of species concurrently; c) predict how future changes in fire regimes might influence community diversity; and d) through the use of traits and not species, compare relationships between bats and fire across regions and internationally.

## CONFLICT OF INTEREST

None declared.

## AUTHOR CONTRIBUTIONS

Rachel V. Blakey developed the paper concept, wrote the document, conducted analysis, and produced figures (with illustrations provided by Lauren Helton, see Acknowledgements). Elisabeth B. Webb & Dylan C. Kesler secured funding, conceptualized the experimental and fieldwork design, developed the paper concept, and provided editorial and scientific input on the manuscript. Rodney B. Siegel developed the paper concept and provided editorial and scientific input on the manuscript. Derek Corcoran supervised field data collection and provided editorial input on a draft version. Matthew Johnson provided funding, scoping and development of overarching project, and editorial and scientific input on the manuscript.

## Data Availability

Bat detection data are archived on the USDA Forest Service Nature Resource Information System (https://www.fs.fed.us/nrm/index.shtml).
