# Supplementary material for: Bats in a changing landscape: Linking occupancy and traits of a diverse montane bat community to fire regime
Source: Ecol Evol. 2019 Apr 12;9(9):5324–37. doi: 10.1002/ece3.5121 (PMC6509396; doi:10.1002/ece3.5121)
Supplement: Supplementary file 1 [file ECE3-9-5324-s001.docx]

**Appendices**

Appendix S1. Summary of all models of occupancy (*ψ*) of 9 bat species in Plumas National Forest, 2015-2017. Detection (ρ) and occupancy (ψ) covariates are listed with degrees of freedom (df), Akaike’s Information Criterion adjusted for small samples (AIC*_c_*), difference of AICc between a model and the model with the lowest AICc (ΔAIC*_c_*), and model weights (ω*_i_*). Model variables included detection covariates ρ() and occupancy variables ψ(), including physiographic, forest structure and fire regime variables. Detection covariates were maximum daily temperature (tmax), minimum daily temperature (tmin), moon illuminated fraction (moon.frac) and moon illuminance (moon.illum). Physiographic variables were: elevation, slope, percentage rock (pc.rock), distance to perennial stream (dist.per), distance to all streams (dist.strm) and distance to all water (dist.water). Forest structure variables included: canopy cover (canopy.cover), mean stand tree diameter (mean.diam), basal area (basal.area), trees per ha (trees.per.ha), distance to open area (dist.open), distance to edge (dist.edge). Fire regime variables included: years since fire (years.since.fire), canopy burn severity (burn.severity), fire return interval continuous (FRI) and categorical (FRI.fac). Temporal variables included: day of season (day.season) and year.

|  | df | AIC*_c_* | ΔAIC*_c_* | ω*_i_* |
| --- | --- | --- | --- | --- |
| *Myotis yumanensis* | |  |  |  |
| ρ(tmax) ψ(elevation mean.diam pc.rock) | 6 | 132.8 | 0 | 0.40 |
| ρ(tmax) ψ(elevation mean.diam pc.rock FRI) | 7 | 134.7 | 1.9 | 0.15 |
| ρ(tmax) ψ(elevation mean.diam pc.rock years.since.fire) | 7 | 134.9 | 2.1 | 0.14 |
| ρ(tmax) ψ(elevation mean.diam pc.rock burn.severity) | 7 | 135.0 | 2.2 | 0.13 |
| *Myotis californicus* | |  |  |  |
| ρ(tmax) ψ(basal.area day.season elevation trees.per.ha pc.rock years.since.fire) | 8 | 292.6 | 0 | 0.27 |
| ρ(tmax) ψ(basal.area day.season elevation trees.per.ha pc.rock FRI) | 8 | 293.2 | 0.5 | 0.21 |
| ρ(tmax) ψ(basal.area day.season elevation trees.per.ha pc.rock) | 7 | 293.5 | 0.9 | 0.17 |
| ρ(tmax) ψ(basal.area day.season elevation trees.per.ha pc.rock burn.severity) | 8 | 295.9 | 3.3 | 0.17 |
| *Myotis lucifugus* | |  |  |  |
| ρ(tmax tmin) ψ(elevation trees.per.ha pc.rock years.since.fire) | 8 | 165.9 | 0 | 0.36 |
| ρ(tmax tmin) ψ(elevation trees.per.ha pc.rock FRI) | 8 | 167.3 | 1.3 | 0.19 |
| ρ(tmax tmin) ψ(elevation trees.per.ha pc.rock) | 7 | 169.1 | 3.2 | 0.07 |
| ρ(tmax tmin) ψ(elevation trees.per.ha pc.rock burn.severity) | 8 | 169.2 | 3.2 | 0.07 |
| *Myotis evotis* |  |  |  |  |
| ρ(tmax) ψ(elevation years_since_fire) | 5 | 303.5 | 0 | 0.33 |
| ρ(tmax) ψ(elevation FRI) | 5 | 304.0 | 0.4 | 0.26 |
| ρ(tmax) ψ(elevation) | 4 | 306.1 | 2.5 | 0.09 |
| ρ(tmax) ψ(elevation burn.severity) | 5 | 307.1 | 3.6 | 0.05 |
| *Eptesicus fuscus* | |  |  |  |
| ρ(tmax) ψ(canopy.cover dist.water mean.diam years.since.fire years.since.fire^2^) | 8 | 221.2 | 0 | 0.85 |
| ρ(tmax) ψ(canopy.cover dist.water mean.diam) | 6 | 226.3 | 5.1 | 0.07 |
| ρ(tmax) ψ(canopy.cover dist.water mean.diam FRI) | 7 | 228.2 | 7.1 | 0.02 |
| ρ(tmax) ψ(canopy.cover dist.water mean.diam burn.severity) | 7 | 228.6 | 7.4 | 0.02 |
| *Lasionycteris noctivagans* | |  |  |  |
| ρ(tmax) ψ(basal.area canopy.cover dist.per) | 6 | 257.7 | 0 | 0.41 |
| ρ(tmax) ψ(basal.area canopy.cover dist.per FRI.fac) | 8 | 259.5 | 1.8 | 0.17 |
| ρ(tmax) ψ(basal.area canopy.cover dist.per years.since.fire) | 7 | 260.0 | 2.2 | 0.13 |
| ρ(tmax) ψ(basal.area canopy.cover dist.per burn.severity) | 7 | 260.1 | 2.4 | 0.12 |
| *Myotis thysanodes* | |  |  |  |
| ρ(.) ψ(canopy.cover pc.rock slope) | 5 | 165.1 | 0 | 0.33 |
| ρ(.) ψ(canopy.cover pc.rock slope burn.severity) | 6 | 165.5 | 0.4 | 0.27 |
| ρ(.) ψ(canopy.cover pc.rock slope FRI) | 6 | 166.7 | 1.6 | 0.15 |
| ρ(.) ψ(canopy.cover pc.rock slope years.since.fire) | 6 | 167.4 | 2.2 | 0.11 |
| *Tadarida brasiliensis* | |  |  |  |
| ρ(tmax moon.frac) ψ(canopy.cover trees.per.ha FRI.fac) | 8 | 196.8 | 0 | 0.42 |
| ρ(tmax moon.frac) ψ(canopy.cover trees.per.ha) | 6 | 197.9 | 1.0 | 0.25 |
| ρ(tmax moon.frac) ψ(canopy.cover trees.per.ha years_since_fire) | 7 | 199.7 | 2.9 | 0.10 |
| ρ(tmax moon.frac) ψ(canopy.cover trees.per.ha burn.severity) | 7 | 199.8 | 2.9 | 0.10 |
| *Lasiurus cinereus* | |  |  |  |
| ρ(.) ψ(canopy.cover) | 5 | 182.3 | 0 | 0.32 |
| ρ(.) ψ(canopy.cover burn.severity) | 6 | 183.4 | 1.0 | 0.19 |
| ρ(.) ψ(canopy.cover years.since.fire) | 6 | 183.5 | 1.2 | 0.17 |
| ρ(.) ψ(canopy.cover FRI) | 6 | 184.3 | 1.9 | 0.12 |

Appendix S2. Model-averaged coefficient estimates and standard errors (SE), with test statistics (z) and p values of top ranking models predicting occupancy of 9 bat species in Plumas National Forest, 2015-2017. Variables are defined in Appendix S1.

| Variable | Coefficient | SE | z | *p* |
| --- | --- | --- | --- | --- |
| *Myotis yumanensis* |  |  |  |  |
| *ψ*(intercept) | -1.11 | 0.42 | -2.63 | 0.009 |
| elevation | -0.74 | 0.43 | -1.73 | 0.084 |
| mean.diam | 1.07 | 0.45 | 2.39 | 0.017 |
| pc.rock | 0.95 | 0.45 | 2.09 | 0.036 |
| *ρ*(intercept) | -0.92 | 0.40 | -2.27 | 0.023 |
| tmax | 1.69 | 0.50 | 3.37 | 0.001 |
| *Myotis californicus* |  |  |  |  |
| *ψ*(intercept) | 2.49 | 0.57 | 4.38 | <0.001 |
| basal.area | 1.76 | 0.89 | 1.97 | 0.048 |
| day.season | 0.72 | 0.42 | 1.7 | 0.089 |
| elevation | -1.01 | 0.49 | -2.06 | 0.039 |
| trees.per.ha | -0.81 | 0.45 | -1.79 | 0.074 |
| pc.rock | -0.66 | 0.35 | -1.9 | 0.058 |
| *ρ*(intercept) | 1.18 | 0.17 | 6.91 | < 0.001 |
| *Myotis lucifugus* |  |  |  |  |
| *ψ*(intercept) | -0.90 | 0.43 | -2.07 | 0.038 |
| elevation | 1.32 | 0.51 | 2.58 | 0.010 |
| trees.per.ha | -0.93 | 0.46 | -2.01 | 0.045 |
| pc.rock | 1.15 | 0.45 | 2.58 | 0.010 |
| years.since.fire | -0.86 | 0.39 | -2.20 | 0.028 |
| *ρ*(intercept) | -0.17 | 0.31 | -0.54 | 0.591 |
| tmax | 1.46 | 0.39 | 3.71 | < 0.001 |
| tmin | -0.61 | 0.35 | -1.75 | 0.079 |
| *Myotis evotis* |  |  |  |  |
| *ψ*(intercept) | 2.05 | 0.52 | 3.92 | < 0.001 |
| elevation | 1.50 | 0.42 | 3.58 | < 0.001 |
| years_since_fire | -0.96 | 0.54 | -1.78 | 0.074 |
| *ρ*(intercept) | 0.34 | 0.16 | 2.09 | 0.036 |
| tmax | 0.71 | 0.18 | 3.92 | < 0.001 |
| *Eptesicus fuscus* |  |  |  |  |
| *ψ*(intercept | 0.12 | 0.42 | 0.29 | 0.768 |
| canopy.cover | -1.61 | 0.68 | -2.36 | 0.018 |
| dist.water | 1.70 | 0.61 | 2.79 | 0.005 |
| mean.diam | 1.22 | 0.49 | 2.51 | 0.012 |
| years.since.fire | -1.36 | 3.87 | -0.35 | 0.725 |
| years.since.fire^2^ | -14.08 | 6.16 | -2.28 | 0.022 |
| *ρ*(intercept) | 0.19 | 0.22 | 0.84 | 0.404 |
| tmax | 0.89 | 0.27 | 3.35 | 0.001 |
| *Lasionycteris noctivagans* |  |  |  |  |
| *ψ*(intercept) | 0.31 | 0.30 | 1.03 | 0.301 |
| basal.area | -0.53 | 0.36 | -1.48 | 0.140 |
| canopy.cover | -1.17 | 0.37 | -3.17 | 0.002 |
| dist.per | -0.48 | 0.33 | -1.46 | 0.144 |
| *ρ*(intercept) | 0.72 | 0.20 | 3.53 | < 0.001 |
| tmax | 0.50 | 0.22 | 2.30 | 0.022 |
| *Myotis thysanodes* |  |  |  |  |
| *ψ*(intercept) | -1.04 | 0.40 | -2.59 | 0.010 |
| canopy.cover | 0.56 | 0.37 | 1.50 | 0.134 |
| pc.rock | 0.78 | 0.46 | 1.70 | 0.088 |
| slope | -1.28 | 0.48 | -2.64 | 0.008 |
| *ρ*(intercept) | -0.48 | 0.35 | -1.35 | 0.176 |
| *Tadarida brasiliensis* |  |  |  |  |
| *ψ*(intercept) | -0.29 | 0.34 | -0.84 | 0.398 |
| canopy.cover | -1.56 | 0.46 | -3.40 | 0.001 |
| trees.per.ha | -0.64 | 0.41 | -1.56 | 0.119 |
| *ρ*(intercept) | 0.17 | 0.24 | 0.71 | 0.475 |
| moon.frac | -0.46 | 0.24 | -1.88 | 0.060 |
| tmax | 0.85 | 0.30 | 2.82 | 0.005 |
| *Lasiurus cinereus* |  |  |  |  |
| *ψ*(intercept) | -0.14 | 0.47 | -0.29 | 0.770 |
| canopy.cover | -1.13 | 0.44 | -2.6 | 0.009 |
| *ρ*(intercept) | -1.01 | 0.31 | -3.21 | 0.001 |
| moon.illum | 0.38 | 0.26 | 1.49 | 0.137 |
| tmax | 0.46 | 0.28 | 1.68 | 0.093 |
